# The highly improved genome of *Ixodes scapularis* with X and Y pseudochromosomes

**DOI:** 10.26508/lsa.202302109

**Published:** 2023-10-09

**Authors:** Andrew B Nuss, Johnathan S Lomas, Jeremiah B Reyes, Omar Garcia-Cruz, Wenlong Lei, Arvind Sharma, Michael N Pham, Saransh Beniwal, Mia L Swain, Molly McVicar, Isaac Amankona Hinne, Xingtan Zhang, Won C Yim, Monika Gulia-Nuss

**Affiliations:** 1 Department of Biochemistry and Molecular Biology, The University of Nevada , Reno, NV, USA; 2 Department of Agriculture, Veterinary, and Rangeland Sciences, The University of Nevada , Reno, NV, USA; 3 Nevada Bioinformatics Center, University of Nevada , Reno, NV, USA; 4 Shenzhen Branch, Guangdong Laboratory for Lingnan Modern Agriculture, Genome Analysis Laboratory of the Ministry of Agriculture, Agricultural Genomics Institute at Shenzhen, Chinese Academy of Agricultural Sciences, Shenzhen, China; 5 Department of Computer Science and Engineering, The University of Nevada , Reno, NV, USA

## Abstract

*Ixodes scapularis*, the black-legged tick, is the principal vector of the Lyme disease spirochete, *Borrelia burgdorferi*, and is responsible for most of the ∼470,000 estimated Lyme disease cases annually in the USA. *Ixodes scapularis* can transmit six additional pathogens of human health significance. Because of its medical importance, *I. scapularis* was the first tick genome to be sequenced and annotated. However, the first assembly, *I. scapularis* Wikel (IscaW), was highly fragmented because of the technical challenges posed by the long, repetitive genome sequences characteristic of arthropod genomes and the lack of long-read sequencing techniques. Although *I. scapularis* has emerged as a model for tick research because of the availability of new tools such as embryo injection and CRISPR-Cas9-mediated gene editing yet the lack of chromosome-scale scaffolds has slowed progress in tick biology and the development of tools for their control. Here we combine diverse technologies to produce the *I. scapularis* Gulia-Nuss (IscGN) genome assembly and gene set. We used DNA from eggs and male and female adult ticks and took advantage of Hi-C, PacBio HiFi sequencing, and Illumina short-read sequencing technologies to produce a chromosome-level assembly. In this work, we present the predicted pseudochromosomes consisting of 13 autosomes and the sex pseudochromosomes: X and Y, and a markedly improved genome annotation compared with the existing assemblies and annotations.

## Abstract (precis)

*Ixodes scapularis* Gulia-Nuss (IscGN) genome assembly and gene set were obtained using DNA from eggs and male and female adult ticks. By combining Hi-C, PacBio HiFi, and Illumina sequencing technologies, we assembled 13 pseudoautosomes and sex chromosomes: X and Y.

## Introduction

A complete genome assembly is required to understand the unique aspects of tick biology and to develop control strategies to reduce their capacity to spread a wide variety of pathogens. The *Ixodes scapularis* genome is relatively large among arthropods (∼2.1–2.5 Gbp) and is highly repetitive, making it challenging to assemble. The first attempt at sequencing a tick genome was initiated in 2008 using embryos from the Wikel strain of *I. scapularis* Wikel (IscaW1). The IscaW1 genome was eventually published in 2016 (Gulia-Nuss et al, 2016). This assembly was highly fragmented (total number of scaffolds = 369,495) and suffered from short contigs (contig N50, 2,942 bp, meaning that only half of the assembly was found on contigs >3 kb) and a total length of combined scaffolds (including gaps) of 1.8 Gbp. The genome was sequenced using Bacterial Artificial Chromosomes clones and Sanger sequencing methods. Unfortunately, some repetitive regions were too large and difficult to be integrated into the available clone libraries, resulting in a fragmented genome. However, the publication of *I. scapularis* genome started the momentum that led to several other chelicerate genome projects, including mite (Dong et al, 2018; Techer et al, 2019) and the *I. scapularis* cell line genome (Miller et al, 2018) followed by six other tick genomes (Jia et al, 2020). A new *I. scapularis* genome assembly was recently published (De et al, 2023) using Hi-C and Pacific Biosciences (PacBio) long reads and was able to provide a high-quality genome assembly. However, further improvement in assembly is needed especially the phasing of sex pseudochromosomes.

Here, we describe an assembly of a high-quality reference genome, *I. scapularis* Gulia-Nuss laboratory (IscGN), that combines long-read PacBio HiFi (Ardui et al, 2018), CHiCAGO and Hi-C (high-throughput sequencing methods based on chromosome conformation capture) (Cairns et al, 2016), and Illumina short-read sequencing technologies. The new high-quality genome assembly now consists of 15 pseudochromosomes, corresponding to 13 pseudoautosomes and X and Y pseudochromosomes. More protein-coding genes have been identified than previous assemblies (35,028 in IscGN compared with 20,486 in IscaW1 and 34,235 in De et al [2023] genome). We curated genes in large multi-gene families that encode chemosensory genes, proteases and protease inhibitors, and cuticular proteins. We also show Hox cluster on pseudochromosome 1.

The IscGN is the first tick genome assembly with phased pseudo-sex chromosome Y. This new genome assembly facilitates an understanding of how ticks parasitize and transmit pathogens to their vertebrate hosts and identifying genes linked to X and Y pseudochromosomes to explore novel tick control methods.

## Results

### Chromosome-scale genome assembly

We present a chromosome-scale 2.47 Gbp assembly of the *I. scapularis* genome with differentiated X and Y pseudochromosomes. A total of 5.38 million PacBio HiFi reads were generated from high molecular weight (HMW) DNA extracted from adult male and female ticks. These long-read sequencing data were used to create an initial contig-level assembly with a sequencing coverage of 26.3X (Table S1). The resulting contig-level assembly was split into 69,985 contigs with N50 of 51.06 kb (Table S1). This assembly spanned 2.95 Gbp of sequence, 476 Mbp more than the final assembly size (Tables S2 and S3). The longer length of the initial contig-level assembly reflects the substantial allelic variation and repeat content of the *I. scapularis* genome. To overcome these challenges, we used the Khaper algorithm, which effectively solved a highly heterozygous diploid tea plant genome (Zhang et al, 2021), to differentiate primary contigs from allelic contig pairs generated from loci with unique haplotypes. The contigs belonging to the X and Y pseudochromosomes were determined using a read-depth strategy that compared the alignment rate of sequencing reads generated from male and female ticks. The Y pseudochromosome was assembled with contigs that were exclusively mapped by reads generated from the male tick-derived DNA. The Illumina paired-end sequencing reads were then used to identify and correct sequencing errors.

Table S1. Genome assembly statistics for the *I. scapularis* assemblies and the assemblies for seven related tick species and two arachnids.

Table S2. Number of contigs per pseudochromosome.

Table S3. Assembly quality statistics for the contig-level assembly and the scaffold-level assembly.

**Figure 1.**
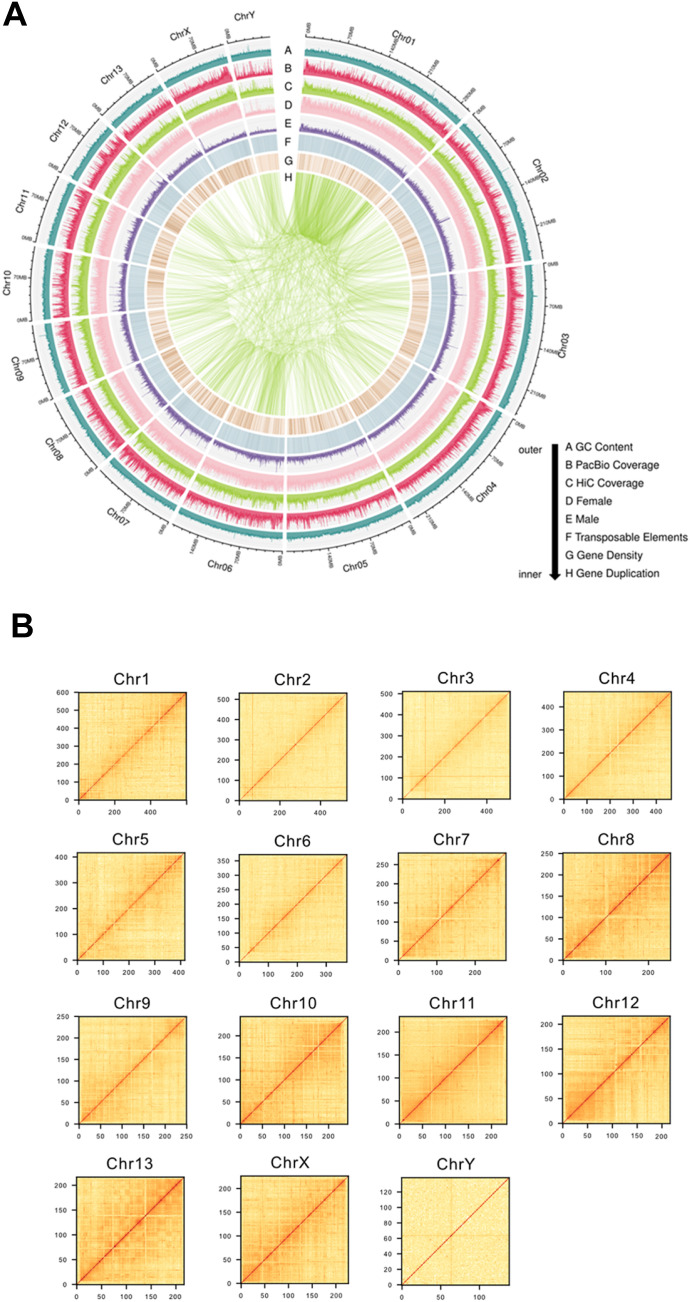
*Ixodes scapularis* genome assembly. **(A)** Chromosomal features of the *I. scapularis* Gulia-Nuss genome assembly. The *I. scapularis* genome is comprised of 13 autosomal chromosomes and two sex chromosomes (X and Y). The tracks, from outer to inner, represent GC % (A), PacBio (B) and Hi-C (C) sequencing coverage, female and male sequencing read depth (D, E), transposable element content (F), and gene density (G). The innermost links connect duplicated genes throughout the genome (H). All plots were drawn on a 100-Kb sliding window. **(B)** Hi-C contact matrices of the *I. scapularis* Gulia-Nuss genome assembly. Loci contact frequency matrices were generated using Hi-C sequencing data and 500 kb bins. The Hi-C matrix for each chromosome is shown as a heatmap with colors ranging from yellow to red with increasing contact frequencies.

**Figure 2.**
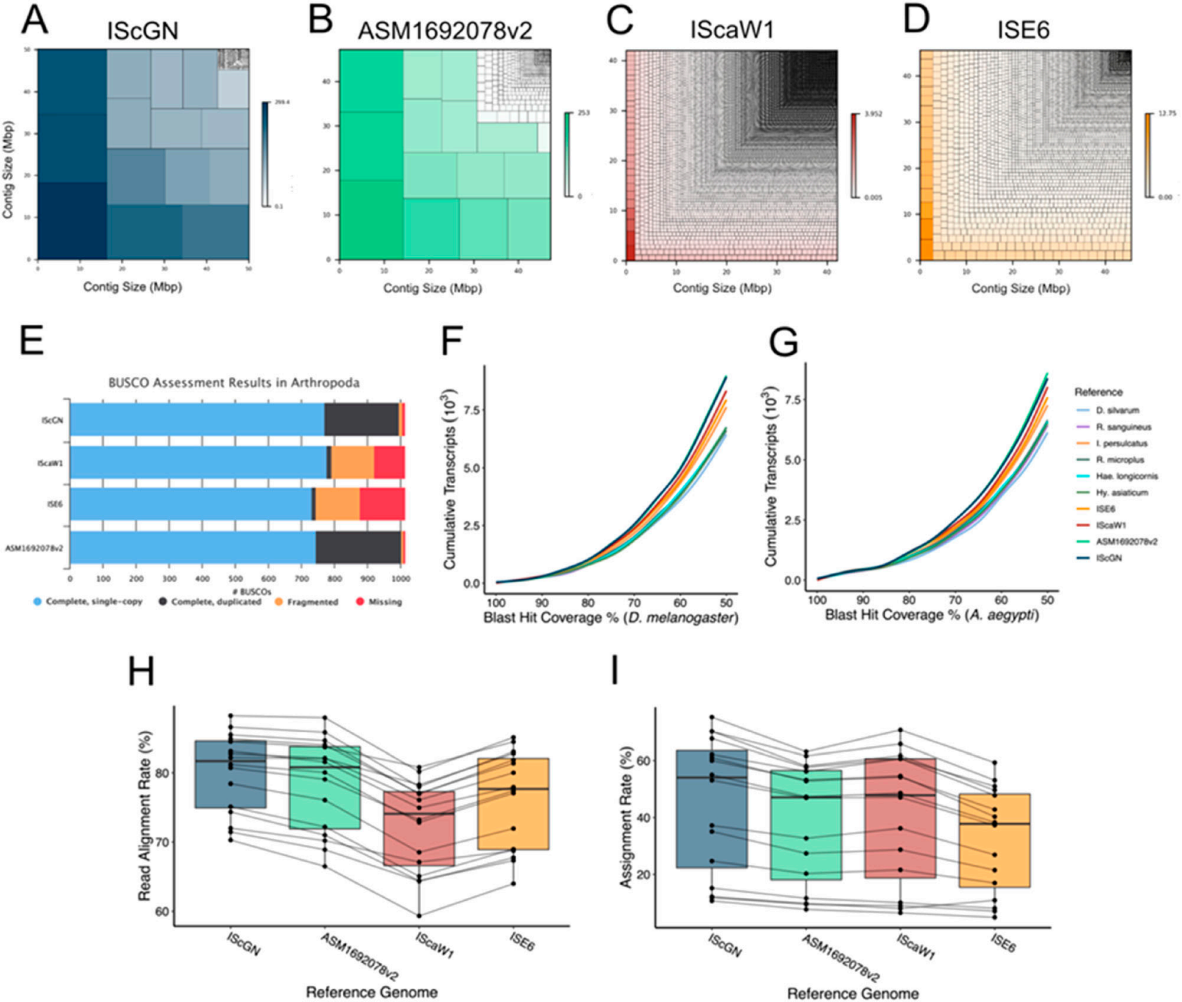
Improvements of the *I. scapularis* genome assembly and annotation. **(A, B, C, D)** The scaffold size distribution of each *I. scapularis* genome assembly is depicted in treemap diagrams (A, B, C, D). **(A)** The presented genome assembly, *I. scapularis* Gulia-Nuss (A), contains fewer scaffolds than any competing assembly. The area of each rectangle represents the size (Mbp) of each chromosome in the assembly. **(E)** Genome completeness was assessed among all *I. scapularis* genome assemblies using the Benchmarking Universal Single-Copy Ortholog Arthropoda dataset (E). Single-copy genes from phylum Arthropoda were used to determine the completeness of tick genome assemblies. **(F, G)** Each figure represents the cumulative number of transcripts at or above each level of Basic Local Alignment Search Tool hit query coverage against vinegar fly, *Drosophila melanogaster* (F), and mosquito, *Aedes aegypti* (G) genomes. **(H, I)** The quality of each genome assembly was assessed for the performance of RNA-seq read mapping (H) and assignment (I). RNA-Seq reads from multiple *I. scapularis* tissue types were aligned to each reference genome with STAR and gene-wise read counts were computed with FeatureCounts to compare the performance of each genome against actual data.

We used the HiRise pipeline (Putnam et al, 2016) to generate a chromosome-level assembly from the chromatin confirmation data to improve the contig-level assembly. The CHiCAGO (in vitro chromatin assay) and Hi-C (in situ chromatin assay) sequencing reads were generated from egg batches of three individual ticks. Each library produced 153 million reads of 2 × 151 bp length sequences. Together, these CHiCAGO library reads provided 25.72 x physical coverage of the genome. The Hi-C libraries generated an average of 142.7 million reads with a 686.8X sequencing depth. The CHiCAGO-based HiRise assembly resulted in an N50 of 419 kb. A total of 50,261 contigs, or 83.76% of the contig sequences, were successfully anchored using CHiCAGO and Hi-C analysis. The final assembly produced an impressive scaffold N50 value of 207.9 Mbp (Table 1), with pseudochromosome 1 representing the largest scaffold at 299.2 Mbp (Tables 1 and S2). A total of 15 pseudochromosomes represent the 13 autosomes and the X and Y sex pseudochromosomes (Fig 1A and B and Tables S2 and S3). Genome-wide analysis of chromatin interactions shows well-organized sequences, supporting a high-quality genome assembly (Fig 1B). The final assembly shows a significant reduction in the number of scaffolds compared with existing *I. scapularis* assemblies (Table S1 and Fig 2A–D). Analysis of the pseudo-sex chromosomes revealed that the 116.4 Mbp predicted X pseudochromosomes is 1.7-fold larger than the 69.3 Mbp predicted Y pseudochromosome, which is the shortest of the *I. scapularis* chromosomes (Table S2). These data agree with previous cytogenetics work in *I. scapularis*, which identified the Y chromosome as the shortest element in the karyotype and suggested that the X chromosomes are much larger than the Y (Chen et al, 1994).

**Table 1.** Statistics of *Ixodes scapularis* genome assembly, IscGN.


Number of pseudomolecule	15
Total size of pseudomolecule (bp)	2,471,497,855
Pseudomolecule N50 (bp)	207,949,265
Number of scaffolds	585
Total size of scaffold (bp)	2,510,615,084
Scaffold N50 (bp)	208,134,562
Number of contigs	69,985
Total size of contig (bp)	2,947,858,410
Contig N50 (bp)	51.06
Longest pseudomolecule	299,173,329
G+C contents (%)	45.9
Repeat content	
Class I transposons	19.3%
Class II transposons	34.7%
Unclassified repeats	3.3%
Repeat contents	57.3%
Annotation	
Number of genes	33,236
Number of predicted transcripts	35,041
Exons	224,465
Introns	189,424
5′ UTR	4,941
3′ UTR	4,022
Benchmarking Universal Single-Copy Ortholog completeness (%)	97.5%

The assembly quality was assessed by the proportion of identified BUSCOs (Benchmarking Universal Single-Copy Orthologs) (Simão et al, 2015). A total of 97.5% of the 2,934 Arachnida lineage BUSCOs were identified, indicating a high-quality genome assembly and annotation (Fig 2E and Table S4). Whereas the number of complete BUSCOs is slightly lower than the annotation of De et al (2023) (ASM1692078v2, 98.9%), both annotations are highly complete and represent a marked improvement from the previous *I. scapularis* assemblies (IscaW1 and ISE6), which contain a much larger proportion of fragmented and missing BUSCOs (Fig 2E). The cumulative Basic Local Alignment Search Tool (BLAST) hit coverage of predicted proteins against the mosquito, *Aedes aegypti*, and vinegar fly, *Drosophila melanogaster*, supports the completeness of tick genome assemblies (Fig 2F and G). Each genome assembly was assessed for the performance of RNA-Seq read mapping (Fig 2H) and assignment (Fig 2I). RNA-Seq reads from multiple *I. scapularis* life stages (eggs, larvae, nymphs, and adult males and females) and tissues (midgut and epidermis) were aligned to each reference genome with STAR (Dobin et al, 2013) and GeneWise (Birney et al, 2004). Read counts were computed with FeatureCounts (Liao et al, 2014) to compare the performance of each genome. In both read alignment and read assignment, IscGN outperformed all other genome assemblies (Fig 2H and I), including the De et al (2023) annotation (Fig 2I). On average, 12% and 4% more RNA-seq reads map to the IscGN gene set annotation than IscaW1 and De et al (2023), respectively (Table S5). Therefore, the IscGN gene set is considerably more complete and correct than previous versions available on VectorBase.

Table S4. BUSCO identification statistics for our IscGN genome assembly compared to previous *I. scapularis* genome assemblies.

Table S5. Improved alignment of RNAseq data.

### Transposable elements

**Figure 3.**
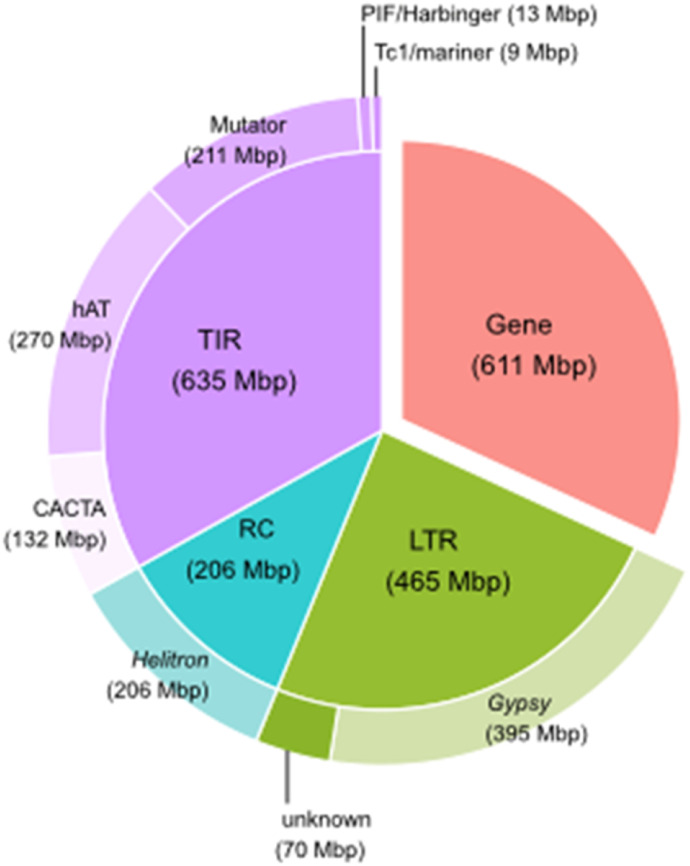
Transposable element families in the *I. scapularis* Gulia-Nuss genome. The distribution of repetitive elements in the *I. scapularis* genome is presented as a proportion of the total length of identified genomic features.

Highly contiguous genome assemblies more accurately reveal the transposable element (TE) content of genomes. Our analysis revealed 1.41 Gbp of repetitive sequence, comprising 57.27% of the assembly (Table S6). The TE content of the individual pseudochromosomes ranged from 54.0–61.42%. The shortest chromosome, pseudochromosome Y, has the highest TE content (Fig 1 and Table S6). On the whole-genome scale, the most prevalent elements were the *Gypsy* LTR retrotransposons, comprising 15.16% of the genome (Fig 3 and Table S7). The *Copia* LTR elements are much scarcer and only constitute 0.02% of the total haploid genome, displaying differential evolutionary pressures on the two LTR superfamilies. A total of 3.21% of the identified LTR lacked a classification, supporting the hypothesis that the arthropod mobilome is more expansive than that of better characterized groups, such as vertebrates and plants, and requires further classification (Petersen et al, 2019). The *I. scapularis* genome contains 1.8-fold more DNA transposon sequences than retrotransposon sequences, with *hAT* elements prevailing at 11.6% of the genome (Table S7).

Table S6. Repetitive sequence annotation by chromosome.

Table S7. Repetitive element identification and classification statistics.

### Predicted protein-coding genes

**Figure 4.**
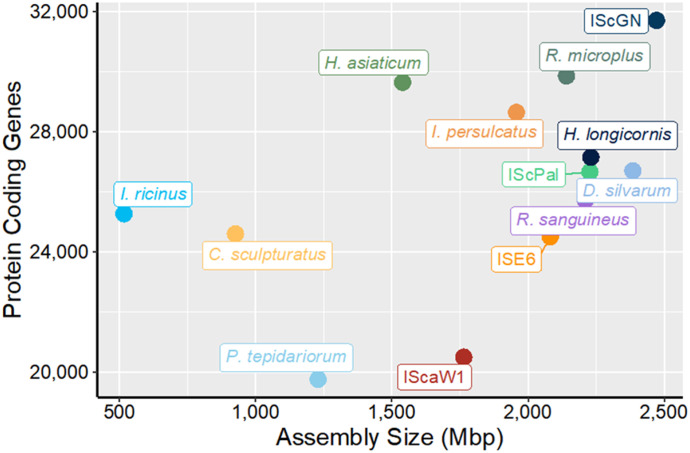
Assembly size versus protein coding gene count among chelicerate genome assemblies. The total number of protein-coding genes detected is plotted against the assembly size. The *I. scapularis* Gulia-Nuss assembly displays significant improvement in the number of protein-coding genes compared with older assemblies.

Complete and correct gene models are essential for studying tick biology. We used the MAKER genome annotation pipeline (Cantarel et al, 2008) to produce an annotation for the IscGN assembly, followed by manual curation of core gene families. A total of 33,236 predicted gene models and 35,041 transcripts were identified in the IscGN genome, comprising 675.7 kb of the total genome (Fig 4 and Table S8). The IscGN assembly formed the basis for a comprehensive quantification of transcript abundance in eight developmental stage-specific, nine midgut timecourse, and three epidermis time-course RNA-seq libraries (Bioproject numbers PRJNA856331, PRJNA1001997; Table S8).

Table S8. Summary of *I. scapularis* gene annotation statistics.

### Curation of multi-gene families

**Figure 5.**
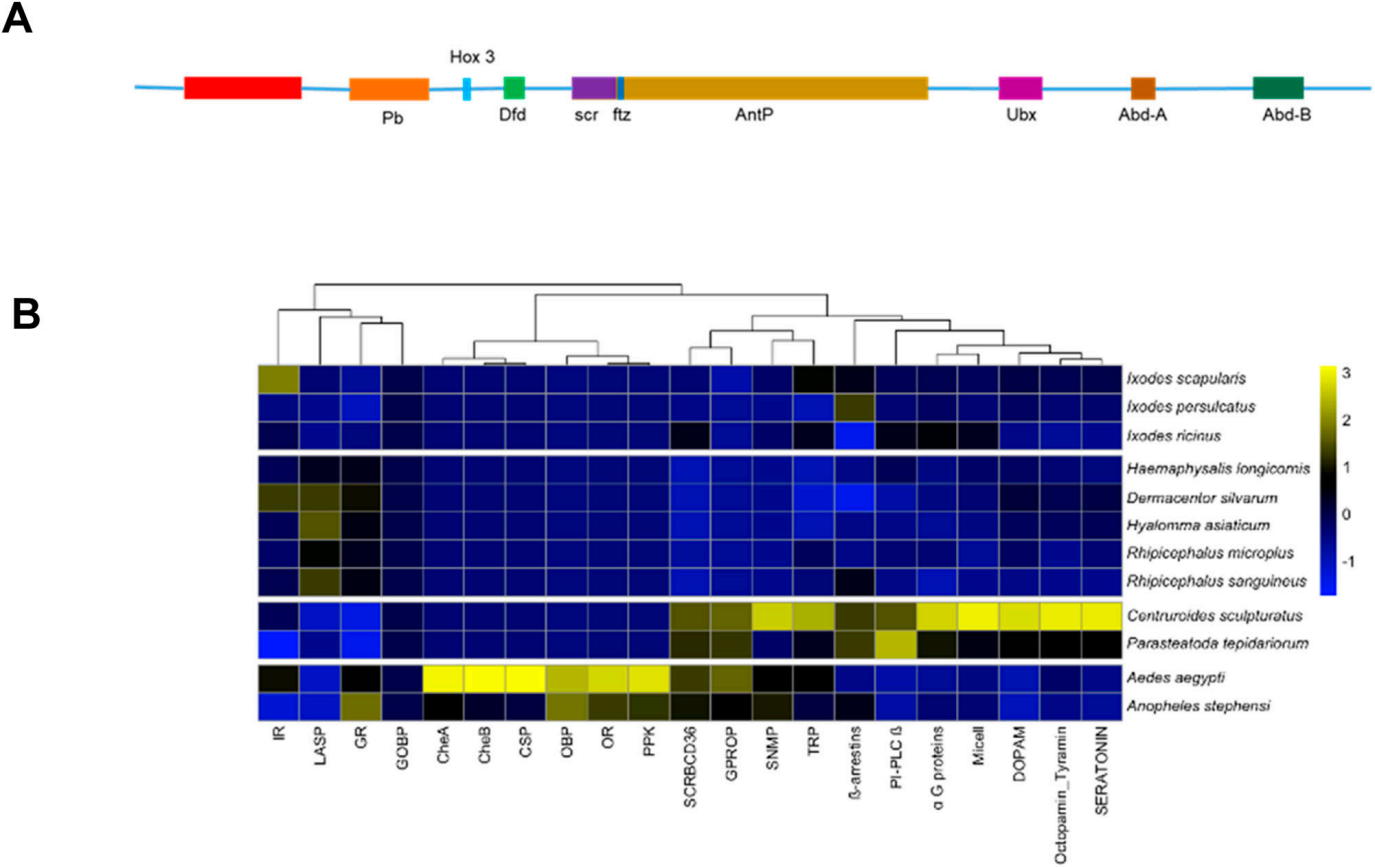
*I. scapularis* Hox genes and chemosensory gene families. **(A)** Genomic organization of Hox genes. Hox genes are clustered on Chromosome 1 on a single genomic scaffold; gene orthology is based on the best Basic Local Alignment Search Tool hit. A colored box represents each Hox gene. The genomic regions containing Hox genes are represented in scale. **(B)** Chemosensory genes among related genomes. Heatmap represents column-wise z-scores of the chemosensory gene counts in each related genome. Gene families and species are organized by hierarchical clustering.

**Figure 6.**
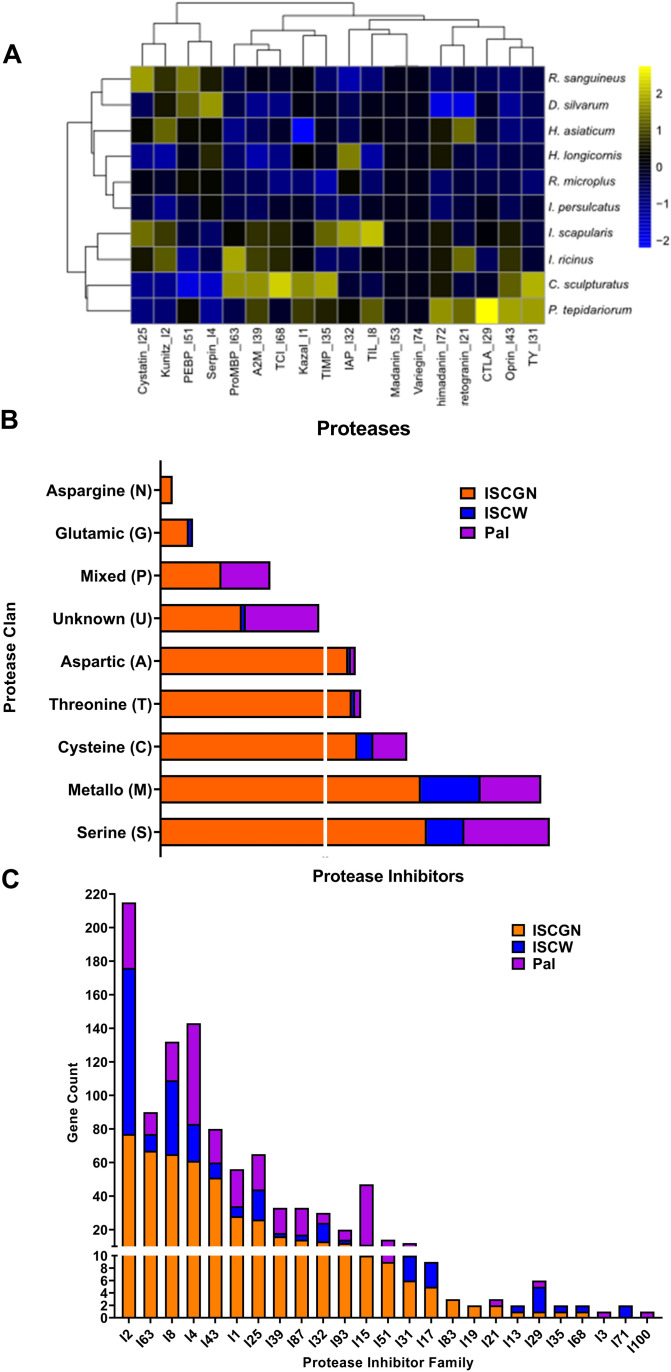
Predicted proteases and protease inhibitors in *I. scapularis* genome. **(A)** Proteases among related genomes. Heatmap represents column-wise z-scores of the protease gene counts in each related genome. Gene families and species are organized by hierarchical clustering. **(B)** Proteases in *I. scapularis* genomes. The vertical white line on X-axis denotes a break in the axis. **(C)** Protease inhibitors in *I. scapularis* genomes. The horizontal white line on Y-axis denotes a break in the axis IscGN, *I. scapularis* Gulia-Nuss assembly; IscaW, *I. scapularis* Wikel genome assembly, Pal, Pal laboratory assembly (De et al, 2023).

Large, multi-gene families are challenging to assemble and correctly annotate because recently duplicated genes typically share high sequence similarities or can be misclassified as alleles of a single gene. We curated genes in large multi-gene families that encode Hox cluster genes, chemosensory genes, peptidases and peptidase inhibitors, and cuticular proteins (Figs 5 and 6).

#### Hox gene cluster

Hox genes encode highly conserved transcription factors in all metazoans that specify the anterior-posterior body axis (Lewis, 1978). Hox genes are clustered in a co-linear arrangement in most vertebrates, whereas in other animal lineages, they are often disorganized (Duboule, 2007). We identified all expected Hox genes in the *I. scapularis* genome clustered on pseudoChromosome 1 (Fig 5A and Table S9), similar to vertebrate genomes.

Table S9. Genomic locations of Hox cluster in *I. scapularis*, *D. melanogaster*, and *A. aegypti*.

#### Chemosensory genes

Three large families of ligand-gated ion channels that act as chemosensory receptors in arthropods were investigated: odorant receptors (ORs), gustatory receptors (GRs), and ionotropic receptors (IRs). These collectively allow arthropods to sense various chemical cues that activate and attract them to food sources, among other functions. From our assembly, we identified 15 GRs and 94 IRs (Fig 5B and Table S10). No ORs were identified in the genome. Because of fragmentation, nine of the 15 GR genes were not detected in the IscaW1 genome assembly.

Table S10. Chemosensory gene counts per family.

#### Proteases and protease inhibitors (PI)

All the major protease and PI families previously reported in arthropods were identified in our genome (Fig 6A). We identified a total of 1,933 putative protease transcripts. Serine proteases comprised most of the proteases (655), followed by metalloproteases (619) (Fig 6B). In contrast, the IscaW1 assembly had 727 predicted proteases, and the majority were metalloproteases (362), followed by serine proteases (228) (Porter et al, 2017). The De et al (2023) assembly had 1,182 total proteases, with the majority being serine (517), followed by metalloproteases (367).

Trypsin-inhibitor-like domain-containing proteins were more abundant in *I. scapularis* compared with other tick species and spiders (Fig 6A). We identified a total of 471 PIs from 22 different families (Fig 6C and Table S11). The largest PI families were I2 (Kunitz-like serine protease inhibitors), I63 (pro-eosinophil major basic protein), I8 (trypsin-inhibitor-like domain elastase inhibitors), I4 (serine protease inhibitors [serpin]), and I43 (oprins, inhibitors of metallopeptidases) (Fig 6C). In comparison, IscaW1 has a total of 244 PIs from 19 different families, and Pal laboratory assembly (De et al, 2023) has a total of 288 PIs from 18 different families.

Table S11. Protease inhibitor gene counts per family.

#### Cuticular/chitin-binding proteins

The arthropod cuticle is primarily formed from two types of structural biopolymers: cuticular proteins (CP) and chitin (Magkrioti et al, 2004). Most CP sequences identified to date from a diversity of arthropods have a conserved region known as the Rebers and Riddiford Consensus (RR Consensus, Gx8Gx7YxAxExGYx7Px2P). Proteins with the RR Consensus (CPR) can be split into three main groups: RR1, RR2, and RR3, depending on the extended N-terminal sequence (Zhao et al, 2017). CPR proteins containing the RR1 motif are mainly found in relatively soft and flexible cuticles, whereas proteins containing the RR2 motif are primarily in hard and rigid cuticles. A few genes encoding CPR proteins with the RR3 motif have been identified in only a few insect species (Dittmer et al, 2015).

A total of 265 CPs were identified in the IscGN assembly. In comparison, 122 CPs were identified in the IscaW1 assembly and 44 CPs in the Pal laboratory assembly (De et al, 2023). Of 265, 93 contained the RR2 motif, compared with 53 in the IscaW1 assembly and 1 in the Pal laboratory assembly (De et al, 2023). Three of the previously identified RR2 motif-containing genes were not supported by our analysis and are not included in the list of 93 genes (Table S12). None of the RR consensus genes showed an RR1 motif, suggesting the hard cuticle of ticks is mainly composed of RR2 motif CPRs.

Table S12. Cuticular/Chitin-binding proteins in IscaW, IscGN, and IscP (I scapularis Pal lab, De et al, 2023) genome assemblies.

Besides CPRs, another major group of CPs is the peritrophin-A motif containing proteins with six distinctly spaced cysteine residues (ChtBD2 domain) (Jasrapuria et al, 2012). This group consists of two proteins with either one or three ChtBD2 domains and is analogous to peritrophins (CPAPs) families 1 and 3. A total of 29 CPAP genes were identified in the IscGN assembly. In comparison, 22 CPAPs were identified in the IscaW1 assembly and 23 in the De et al (2023) assembly.

## Discussion

We present a highly improved reference genome for *I. scapularis*, constructed using modern sequencing technologies, including PacBio HiFi and Hi-C. The assembly is vastly improved compared with previous *I. scapularis* genome assemblies, IscaW1 (Gulia-Nuss et al, 2016), a highly used *I. scapularis* cell line assembly, ISE6 (Miller et al, 2018), and also a newly published, improved assembly ASM1692078v2 (De et al, 2023). Our IscGN genome assembly exhibits greater continuity, consisting of merely 15 pseudochromosomes, in contrast to the ASM1692078v2, IscaW1, and ISE6 genome assemblies, which comprise 648, 369,496, and 6,476 scaffolds, respectively. This assembly is the first to successfully segregate the X and Y pseudochromosomes. It should be noted that sex chromosome pairs (X/Y) may not be assembled with high precision, especially pseudoautosomal regions (Li et al, 2019) that are similar to each other. However, sex chromosomes or their segments can be identified by an outstanding ratio of read coverage between a male and a female when whole genome sequencing reads covering both sexes are available (Palmer et al, 2019). Therefore, our method of using reads from male and female whole genomes and ratios of read coverage is supported by the published literature and provides confidence in phasing sex chromosomes.

Our 2.47 Gbp assembly (predicted 13 autosomes + X + Y) is over 200 Mbp larger than the previous genome size estimates of 2.1 and 2.23 Gbp. These genome sizes were generated from flow cytometry and DNA reassociation kinetics, respectively (Ullmann et al, 2005; Geraci et al, 2007). The increase in genome size observed in our study, relative to previous ones, is likely a product of advanced assembly methodologies used and the enhanced capacity to capture transposable elements, which have been challenging to sequence and assemble using older sequencing technologies. The size of the IscaW1 assembly is an underestimate as the assembly was constructed from Sanger sequencing data and was thus highly fragmented. As for the ISE6 assembly, cell lines can vary in their chromosome numbers after numerous passages (Kotsarenko et al, 2020). Thus, the ISE6 genome may be truly smaller than that of the whole tick because the cell line has been in use since 1994 without any recent karyotyping work (Munderloh et al, 1994). The two older assemblies were also constructed without the chromatin conformation approach, which proved essential for assembling our chromosome-scale *I. scapularis* genome. A significant improvement in the contiguity of the IscGN genome assembly was noticed as a result of the loci contact frequency data.

The size of our assembly is comparable to another *I. scapularis* genome assembly, ASM1692078v2, that was recently published (De et al, 2023). The ASM1692078v2 assembly was constructed using DNA extracted from female ticks and had a final genome size of 2.23 Gbp, which is 280 Mbp smaller than our predicted female tick genome (13 + XX) size of 2.51 Gbp (Table S1). However, they might not have phased out the homologous pair of sex chromosomes. The IscGN assembly with collapsed X pseudochromosomes (13 + X) would be 2.40 Gbp. There might be true variation in the genome sizes among the *I. scapularis* accessions sequenced because the *Ixodidae* family has an estimated average haploid genome size of 2.67 Gbp (Geraci et al, 2007). Another reason for the larger genome size could be the variations in tick populations. Although both labs used the ticks originating from Oklahoma, these ticks were reared in our respective laboratories at UNR and Maryland. The Oklahoma tick lab replenishes colonies with wild-caught ticks and therefore the starting culture may have variations.

This discrepancy in genome size might also be attributed to the repeat content in our genome, which is ∼57.27% compared to 56.47% reported by De et al (2023), a difference of 155 Mbp. The increased repeat content could be because of the possible accumulation of repetitive sequences within the predicted Y chromosome of our assembly (Chang et al, 2022). It has been suggested that Y chromosomes contain a large amount of repeat sequences (Katsura et al, 2012). Our protein-CDS prediction indicated 33,236 protein-CDSs with a mean length of 3.25 kb that give rise to 35,041 transcripts, a higher number than the IscaW1 assembly and the De et al (2023) assembly. Thus, our assembly likely represents a high-quality genome size for *I. scapularis*.

We used PacBio HiFi sequencing and contigs derived from adult males and females, whereas the scaffolding used for CHiCAGO and Hi-C sequencing originated from egg masses. The reason for using disparate tick stages was to initially improve the existing IscaW1 genome assembly, which was derived from eggs. However, the HiC/HiRise assembly still resulted in 83,347 scaffolds (compared with 369,495 scaffolds in IscaW1) (Nuss et al, 2018 *Preprint*). To improve contiguity and in an attempt to identify phased sex chromosomes, we used male (pool of 5) and female (1) DNA samples for PacBio sequencing. We ran the HiRise assembly again using these new DNA sequences, which resulted in 14 C-scaffolds. Whereas the higher order chromatin organization of eggs and adults might differ, in Hi-C scaffolding, the choice of materials is less important because it targets the reconstruction of the whole genome as the uniform goal, even when using different cell populations in an organism. Other studies have suggested that the use of numerous types of tissues may yield optimal performance by covering maximally diverse chromatin contacts (Yamaguchi et al, 2021). Our assembly stands out with the most considerable scaffold N50 value, exceeding the ASM1692078v2 assembly by 75.9 kb, even though both used Hi-C contact frequency data for scaffolding.

The first *I. scapularis* assembly, IscaW1 (Gulia-Nuss et al, 2016), suggested a highly conservative estimate of 16.7% repeat content. Our remarkably contiguous reference genome allowed a more thorough characterization of the *I. scapularis* repetitive sequences and transposable element (TE) repertoire. Our analysis revealed 1.31 Gbp of repetitive sequence, comprising 57.27% of the assembly (Table S6). However, these results are lower than previous estimates. DNA reassociation kinetics analysis by Ullmann et al (2005) estimated a 2.26 Gbp genome with a repeat content of 66.2%. Interestingly, the ISE6 *I. scapularis* cell line has a TE content of 63.5% (Miller et al, 2018), which is ∼6% more than our assembly. The higher TE content of the ISE6 cell line could imply cell line-specific TE dynamics, as *D. melanogaster* cell lines tend to have a higher TE content than whole flies (Rahman et al, 2015; Han et al, 2021). Our comparative genomics analysis included seven tick species and two other chelicerates: *Parasteatoda tepidariorum* (common house spider) and *Centruroides sculpturatus* (bark scorpion) (Schwager et al, 2017). The *I. scapularis* genome is larger and contains more genes than all the species tested (Fig 5 and Table S1). In line with the larger genome size, the *I. scapularis* genome has a higher gene content than the six species analyzed by Jia et al (2020), which predicted an average of 27,566 protein-coding genes (Table S1). Furthermore, arthropod gene content varies considerably, with the sand fly (*Lutzomyia longipalpis*) genome containing 10,110 genes and the pea aphid (*Acyrthosiphon pisum*) genome containing 36,195 genes (Thomas et al, 2020). The *I. scapularis* genome has a lower TE content at 57.3% than the other six species sequenced recently (Jia et al, 2020), with an average TE content of 60.2% (Table S1). Total TE content and relative proportions are highly variable among arthropod genomes, even within orders. Whereas larger arthropod genomes tend to have more repetitive sequences, the correlation has a high range of dispersion, possibly because of population-specific TE activity or segmental duplications and deletions (Petersen et al, 2019). Notably, the *I. scapularis* assembly encompasses approximately three times as many LTR sequences as the *Haemaphysalis longicornis* assembly (Yu et al, 2022).

Genome-wide analysis of chromatin interactions among the final assembly reveals well-organized sequences, suggesting a highly contiguous genome assembly (Fig 1B). In the IscaW1 and ISE6 assemblies, only 1.4% and 1.2% of the BUSCOs were duplicated, whereas 24.3% of the BUSCOs were duplicated in the IscGN assembly. This duplication is in line with the 12,750 more predicted gene models than the previous IscaW1 genome annotation, which only contained 20,486 gene models (Table S1). The chromosome-scale arthropod genome assemblies have revealed a higher percentage of duplicated BUSCOs than less contiguous assemblies (Hotaling et al, 2021; Saha et al, 2021), supporting that IscGN assembly is much higher quality compared with the previous assembly. Interestingly, the De et al (2023) annotation contained ∼7% more duplicated BUSCOs. This disparity could potentially be attributed to the usage of Hifiasm and purge dups during assembly. If the assembly process inadvertently incorporated duplicated haplotypes, it would account for the higher number of duplicated BUSCOs.

Based on the assumption that the female genome contains two X chromosomal copies and lacks Y, whereas the male genome contains one copy each of the X and Y chromosomes, contigs were classified as X-linked if they exhibited twofold copy number variations in females compared with males and as Y-linked if they were detectable in males but absent in females. Although we cannot rule out the possibility that observed copy number variations could be attributed to individual differences rather than gender-based differences, our dataset included genomes from three females (triplicate) and three males, giving us high confidence in predicted male-specific contigs. Once more sequencing data are available, the probability of identifying the male-specific region of the Y chromosome (MSY) based on K-mers would increase. In the absence of this, it is important to acknowledge the limitations inherent in our genome assembly. However, our analysis of chromosome depth revealed a notable decrease in sequencing coverage on the predicted Y chromosome compared with other chromosomes. This finding aligns with previous research (Fig 1) (Chang et al, 2022) and infers support for the accuracy of our chromosome assignment. It also reinforces the notion that the Y chromosome contains a higher proportion of repetitive sequences and heterochromatin, which contribute to the challenges encountered during sequencing (Mahajan et al, 2018). Whereas we successfully attained a chromosome-level assembly for the sex chromosomes, our efforts to definitively identify the Sex Determination Region or MSY and potentially the presence of pseudoautosomal regions, encountered challenges. This work provides us with contig sequences that could be used for developing FISH probes for delineating the MSY.

Our manual curation of multigene families resulted in a higher number of genes in each family than previously published *I. scapularis* genomes, likely because of the contiguous genome and better annotation. In addition, the Homeobox (Hox) gene cluster, responsible for the development of the body plan in animals, was identified on pseudochromosome 1. The arrangement of all Hox genes on one chromosome suggests a co-linear arrangement similar to that of vertebrates. We identified 1,206 additional proteases, 133 additional cuticular/chitin-binding proteins, and nine new GRs. We expect that the improved genome assembly and annotation will spur the identification of genes in other gene families and enhance tick genetics and genomics research.

## Materials and Methods

### Sample collection

Fully engorged *I. scapularis* females were obtained from the Oklahoma State University Tick Rearing Facility, and a colony was maintained at 20°C and 98% relative humidity (RH) in the Gulia-Nuss laboratory. Three batches of eggs of ∼3,000 eggs each were collected from three individual females and immediately flash-frozen in liquid nitrogen. The samples were then shipped to Dovetail Genomics for CHiCAGO and Hi-C library preparation. For the PacBio HiFi sequencing, we used the *I. scapularis* Oklahoma strain reared in our laboratory at UNR. High molecular weight (HMW) DNA was extracted from either a single female or a pool of five males using an HMW extraction protocol adapted from Miller et al (1988).

### CHiCAGO library preparation and sequencing

Three CHiCAGO libraries were prepared as described previously (Putnam et al, 2016). Briefly, ∼500 ng of DNA with a mean fragment length of 100 bp was reconstituted into chromatin in vitro and fixed with formaldehyde. The fixed chromatin was digested with DpnII, the 5′ overhangs filled in with biotinylated nucleotides, and the free blunt ends were ligated. After ligation, crosslinks were reversed, and the DNA was purified from the protein. Purified DNA was treated to remove biotin that was not internal to ligated fragments. The DNA was then sheared to a mean fragment size of ∼350 bp and sequencing libraries were generated using NEB Next Ultra enzymes and Illumina-compatible adapters. Biotin-containing fragments were isolated using streptavidin beads before PCR enrichment of each library. The libraries were sequenced on the Illumina HiSeq X platform using the rapid run mode.

### Hi-C library preparation and sequencing

Three Hi-C libraries were prepared as described previously (Lieberman-Aiden et al, 2009). Briefly, egg tissue was treated with formaldehyde to fix the chromatin in place and the crosslinked chromatin was extracted. The chromatin samples were further prepared and sequenced as for CHiCAGO sequencing, above.

### Contig-level genome assembly

The initial assembly at the contig level was generated using the hifiasm algorithm (Cheng et al, 2021). By implementing this algorithm, we successfully generated a comprehensive set of contig sequences, resulting in a total of 5.3 Gbp of sequences. Upon analyzing the contig sequences, it became apparent that they exceeded the estimated genome size by a significant margin. This discrepancy indicated the presence of a substantial number of redundant sequences within the initial assembly. To address this issue and mitigate the redundancy within the assembly, we used the Khaper algorithm (Zhang et al, 2021). This algorithm is specifically designed to identify and subsequently eliminate redundant sequences using a non-repeat k-mer-based approach. The algorithm selects primary contigs from allelic contig pairs based on their contig length, following the method described by Zhang et al (2021).

### Identification of X and Y-linked contigs

Contigs associated with the Y-chromosome were identified through a read-depth-based strategy, with the objective of identifying contigs present in male-derived reads but absent in female-derived reads. This process involved mapping PacBio HiFi reads from female and male samples against the assembled contigs, facilitating the computation of genome-wide read coverage. In addition, the copy number for each contig was determined using popCNV (https://github.com/sc-zhang/popCNV), a software that uses a concept similar to a previously established method (Bickhart et al, 2012).

Briefly, the software mosdepth (Pedersen & Quinlan, 2018) was used to compute read coverages, which were subsequently adjusted using a locally estimated scatterplot smoothing strategy. This adjustment served to minimize the potentially adverse effects of regions with high or low GC content within each sliding window. The copy numbers were determined based on the ratio of the corrected read depth to the overall sequencing depth of the input fastq file. Contigs present as a single copy in male samples but not detectable in female samples were classified as Y-linked contigs.

To identify X-linked contigs, a similar read-depth-based strategy as that used for Y-linked contigs was used, but with the additional integration of comparative analysis between male and female samples. The aim was to identify contigs with higher copy numbers in female-derived reads as compared with male-derived reads. For the X-linked contigs, the copy numbers were determined based on the ratio of the corrected read depth in female samples to that in male samples. The contigs with corrected read depth ratios indicating an increased copy number in female samples but not deviating significantly from the expected single or fewer copies in male samples were categorized as X-linked contigs.

### Scaffolding the genome assembly with CHiCAGO and Hi-C sequencing

The CHiCAGO and Hi-C read pairs were aligned to the de novo IscGN assembly using a modified SNAP read mapper to identify and correct errors in the scaffolds of the initial (contig-level) *I. scapularis* genome assembly (Zaharia et al, 2011 *Preprint*) (https://www.microsoft.com/en-us/research/project/snap/). The CHiCAGO read pairs that were individually mapped within the draft scaffolds were analyzed by HiRise to produce a likelihood model describing the genomic distance between the read pairs (Putnam et al, 2016). The resulting model was used to identify and break putative misjoins, score prospective joins, and make joins above a specified threshold. After scaffolding by CHiCAGO data, the Hi-C library read pairs were used for the second round of scaffolding. The scaffolds were ordered and oriented using the ALLHiC pipeline, which uses the contact frequency of loci to determine their proximity in the genome (Zhang et al, 2019). Scaffolds less than 100 kb were excluded from the analysis. After scaffolding, shotgun sequences were used to close gaps between contigs. Finally, the contigs and scaffolds that correspond to overlapping regions of the genome were merged by identifying pairs of scaffolds that exhibit both strong sequence homology and strong similarity in long-range contact patterns.

### Polishing draft genome sequences

The finalized assembly of the IscGN genome consisted of 13 pseudoautosomes, in addition to the sex-determining X and Y pseudochromosome scaffolds. An iterative refinement of this chromosomal-scale genome assembly was undertaken with Pilon software (Walker et al, 2014). Before this, the Illumina sequencing reads were subjected to quality control and trimming via Trimmomatic (Bolger et al, 2014). These trimmed reads were then mapped to the preliminary draft of the genome using the Burrows-Wheeler Aligner (Li & Durbin, 2009). The subsequent alignment file was processed through SAMtools (Danecek et al, 2021) and subsequently served as the input for further refinement through Pilon software (Walker et al, 2014).

### Transposon element identification

Repetitive sequences in the IscGN genome assembly were initially discerned via de novo repeat library generation using the Transposable Element (TE) identification package from REPET (Flutre et al, 2011). This package has the capacity to automatically conduct a repeat annotation pipeline that incorporates RECON v1.08 (Bao & Eddy, 2002), BLASTER (Quesneville et al, 2003), GROUPER (Quesneville et al, 2003), and PILER (Edgar & Myers, 2005). After generation, this repeat library underwent annotation by the TE annotation package within REPER, using RepeatMasker (Smit et al, 2013), BLASTER (Quesneville et al, 2003), CENSOR (Jurka et al, 1996), Tandem Repeats Finder (Benson, 1999), MATCHER (Flutre et al, 2011), and mreps (Kolpakov et al, 2003).

For a comprehensive exploration of the repetitive sequences in the *I. scapularis* genome, additional tools were used, including TransposonPSI (http://transposonpsi.sourceforge.net/), MITE-Hunter (Han & Wessler, 2010), LTRharvest (Ellinghaus et al, 2008), and RepeatModeler2 (Flynn et al, 2020). Identified repeat sequences from each species and approach were consolidated, and subsequently, ProtExcluder (Campbell et al, 2014) was used to isolate and exclude portions of the gene sequence corresponding to matches in the UniProt-Swiss database (Boutet et al, 2016).

### Genome annotation

Illumina paired-end RNA-Seq reads were trimmed for low-quality and adaptor sequences using Trimmomatic (Bolger et al, 2014). Subsequently, we aligned the trimmed reads to the genome assembly using HISAT2 (Kim et al, 2019). The mapped reads were provided as input to the BRAKER2 pipeline (Brůna et al, 2021). Within this pipeline, we trained the ab initio gene predictors AUGUSTUS (Stanke et al, 2006) and GeneMark-ET (Besemer & Borodovsky, 2005) to predict complete gene models. In addition, we performed a de novo transcriptome assembly using Trinity (Haas et al, 2013) and RNAseq data.

The MAKER pipeline was run in three iterations to generate a comprehensive genome annotation. First, MAKER was run with ab initio models from BRAKER2, ab initio models generated using FGENESH with a pre-trained *I. scapularis* model (Solovyev et al, 2006), and arthropod protein sequences from SwissProt (Boutet et al, 2016) and TrEMBL (Apweiler et al, 2004) (Taxon ID 6656). Translated proteins derived from the de novo *I. scapularis* transcriptome assembly were also included to provide empirical protein evidence. The resulting gene models were filtered to include those with an Annotation Edit Distance greater than 0.7, which contained a significant (*E*-value < 1 × 10^−10^) PFAM domain identified by HMMER (Eddy, 2011). The filtered gene models were then used to train and predict gene models using SNAP (Korf, 2004) and AUGUSTUS (Stanke et al, 2006). The MAKER pipeline was run a second time using AUGUSTUS, SNAP, FGENESH, and the SwissProt and TrEMBL arthropod protein sequences. Optimal full-length transcripts were subsequently predicted from the combined results of MAKER, AUGUSTUS, and FGENESH using the MIKADO pipeline. This set of full-length transcripts was further filtered to select complete ORFs using Transdecoder (https://github.com/TransDecoder/TransDecoder). A third round of the MAKER pipeline was then run using the MIKADO/Transdecoder protein sequences alongside the arthropod protein sequences from SwissProt and TrEMBL. The resulting gene models were filtered to retain those that were supported by at least 30% AUGUSTUS annotation, expressed at least three FPKM, included a significant (*E*-value < 1 × 10^−10^) PFAM domain, or had 50% BLASTP alignment coverage in either SwissProt or TrEMBL (Taxon ID 6656).

For the newly annotated genes, we adhered to the gene model nomenclature standards for ticks as outlined by VectorBase. These standards involve using three abbreviations derived from the species name, followed by an assigned number consisting of five digits. To differentiate the genes from the previous version, we made a specific modification by removing a single capital letter “W” that was previously present in the gene number. This letter “W” denoted the Wikel strain of *I. scapularis*. Therefore, in the current version, the gene names will be represented as ISCGN instead of ISCW, allowing for a clear distinction.

BUSCO analysis was performed to assess the completeness of gene annotations (Simão et al, 2015). BUSCO v 5.2.2 was run using the protein mode and arachnida_odb10 and arthropoda_odb10 lineage datasets. To provide gene descriptions, Automated Assignment of Human Readable Descriptions (Hallab et al, 2020) was run using independent BLASTp results from SwissProt, TrEMBL, and *Anopheles gambiae*. Known protein domains were identified with InterProScan (Jones et al, 2014), and Gene Ontology (GO) terms were derived from the Assignment of Human Readable Descriptions results.

### Tick samples for RNAseq

Pathogen-free, unfed *I. scapularis* ticks were kept in an incubator at 95% RH and 20°C in our laboratory. Larval and nymphal ticks were blood-fed on mice, and adult ticks were fed on New Zealand white rabbits. All procedures were approved by the Institutional Animal Care and Use Committee at the University of Nevada, Reno (# 21-01-1118; Institutional Animal Care and Use Committee).

### RNA extraction

For midgut transcriptomes, adult females were collected at different time points: unfed (UF), removed from the host 5 d after attachment (partially engorged, PE), and at 1, 7, and 14 d (D) after voluntary host drop-off (post blood meal, PBM). Ticks were surface cleaned with 70% ethanol. Whole midguts were dissected in cold PBS from three ticks and pooled (six for unfed samples) at each time point. Intact midguts were washed in cold PBS to remove blood. Once cleaned of blood, midguts were immediately transferred to a cold 1.7 ml tube containing 200 μl of Trizol and stored at −80°C until processed.

For epidermis/cuticle samples, adult females were collected at UF and PE (3 and 6 d post-attachment time points). Epidermis/cuticles were collected by removing all other tissues and scraping off the epidermal layer from the hard outer cuticle. Three females were pooled per replicate (six for unfed samples). All samples were collected in triplicates.

For developmental stages, 7 d old eggs (∼500 per pool), larvae (UF and 2 D PBM, ∼100 per sample), nymphs (UF, 2D PBM, 20 per sample), adult females (UF, 2 per sample), 1 D PBM (1 per sample), and males (UF, 5 per sample) were collected, surfaced cleaned, and processed in triplicates.

Total RNA was extracted using a Trizol reagent (Invitrogen) and a Zymo Direct-zol kit (Zymo Research).

### Illumina sequencing

Total RNA samples were submitted to either Novogene or Genewiz Inc. for Illumina RNA library construction and sequencing. The mRNA-enriched and amplified library fragments were purified and checked for quality and final concentration using an Agilent 2100 Bioanalyzer with a High Sensitivity DNA Chip (Agilent Technologies). The final quantified libraries were pooled in equimolar amounts for sequencing on an Illumina HiSeq 2500 DNA sequencer, using a 150-bp paired-end sequencing flow cell with a HiSeq Reagent Kit (Illumina).

### Transcriptome analysis

Illumina paired-end RNA-Seq data was acquired and retrieved using the fastq-dump tool (Leinonen et al, 2011). Pre-processing steps were used to enhance data quality, including the trimming of low-quality sequences and adapter sequences using Trimmomatic (Bolger et al, 2014). Subsequently, the trimmed reads were aligned to the reference genome of each respective species using Hisat2 (Kim et al, 2019). The alignment results were then subjected to sorting procedures using SAMtools (Danecek et al, 2021), ensuring appropriate arrangements for downstream analysis. To quantify the gene expression levels, featureCounts (Liao et al, 2014) was used to convert the aligned reads into raw read counts, representing the number of reads mapped to each annotated genomic feature. To enable comparison across samples, these raw read counts were normalized to transcripts per million values, which account for transcript length and library size variations.

### Chemosensory gene sequences

Phylogenetic trees were constructed using predicted chemosensory genes. To analyze the evolutionary relationships among these genes, the corresponding peptide sequences within the orthogroups were aligned using MUSCLE v3.8.31 (Edgar, 2004). Subsequently, the CDSs were aligned onto the amino acid alignments using PAL2NAL v13 (Suyama et al, 2006), allowing for a comparison between nucleotide and protein sequences. Alignments were filtered using TRIMAL (Capella-Gutiérrez et al, 2009), applying two criteria. First, columns in the alignments were removed if gaps were present in more than 90% of the sequences (rows in the alignment matrix). Second, transcript translations were excluded if their coverage accounted for less than 30% of the total alignment length for the gene family. To estimate the gene trees, maximum likelihood (ML) analysis was performed using RAxML v7.3.0 (Stamatakis, 2006) with the general time reversible + Γ substitution model. Bootstrap replicates (n = 1,000) were used to assess the reliability of the inferred phylogenetic relationships.

### Analysis of cuticular proteins

Cuticle proteins (CPs) were identified using eggNOG-mapper (Cantalapiedra et al, 2021) in conjunction with the DIAMOND aligner (Buchfink et al, 2015) to match protein sequences to their functional groups and families. The eggNOG 5.0 database (Huerta-Cepas et al, 2019) provided the Arthropoda orthology and phylogeny group. The additional database included HMMER V3.3.2 (Finn et al, 2011; Potter et al, 2018) to tag for hidden Markov models for identification of chitin-binding (PF00379: CPR) (Mistry et al, 2021) and ExPasy database for chitinase protein identification (EC3.2.1.14) (Gasteiger et al, 2003). FASTA amino acid sequences of the proteins identified as CPs from the IscGN assembly and IscPal assembly (VEuPathDB) (Amos et al, 2022; De et al, 2023) were extracted using the Biopython package (https://github.com/LittleRibosome/GN-Genome-I.-scap-.git). These sequences were analyzed for CPAPs, RR3, RR2, and RR1 extended motifs using the arthropod cuticle database (CutProtFam-Pred [uoa.gr]) (Karouzou et al, 2007; Ioannidou et al, 2014). The cutoff score was 22 or an *E*-value of 5 × 10^−7^ for the respective protein annotation. Cuticle protein annotation from the eggNOG 5.0 (Huerta-Cepas et al, 2019), HMMER, and Expasy databases were added to their respective IscGN or IscPal gene ID. Gene annotations of IscaW and IscGN assemblies were based on NCBI-BLAST+ run using Linux (https://github.com/LittleRibosome/GN-Genome-I.-scap-.git). CPs from the Apl laboratory assembly were similarly matched to the corresponding IscGN using NCBI-BLAST+. Using a cutoff identity score of 75% or bit score <300. VEuPathDB’s annotation, curation, and identifiers tool was used to add an annotation for all IscaW proteins.

### Proteases and protease inhibitors

The amino acid sequence file was analyzed using NCBI-BLAST+ (Camacho et al, 2009) and the MEROPS database (Rawlings et al, 2018) “pepunit.lib” to identify peptidase and inhibitors. MEROPS peptidase and peptidase inhibitor accession numbers identified were matched to their respective peptidase/inhibitor family and clan. All matches were also checked in UniProt (UniProt Consortium, 2021) and HMMER (http://hmmer.org/) for identification of protease and protease inhibitors.

### Genomic data

The following genome sequence and annotation data were downloaded from public repositories (Table S1): *I. scapularis* (release ASM1692078v2) from NCBI, *I. scapularis* (release IscaI1.0 and release IscaW1.7) from VectorBase, *H. asiaticum* (release ASM1333968v1), *H. longicornis* (release ASM1333976v1), *R. microplus* (release ASM1333972v1), *Ixodes persulcatus* (release BMI_IPER_1.0), *R. sanguineous* (release ASM1333969v1), *Drosophila silvarum* (release ASM1333974v1) from NCBI, *A. aegypti* (release AaegL5.3) from VectorBase, and *D. melanogaster* (release dmel_r6.44) from FlyBase.

## Supplementary Material

Reviewer comments
