# Supplementary material for: The highly improved genome of Ixodes scapularis with X and Y pseudochromosomes
Source: Life Sci Alliance. 2023 Oct 9;6(12):e202302109. doi: 10.26508/lsa.202302109 (PMC10561763; doi:10.26508/lsa.202302109)
Supplement: Reviewer comments [file LSA-2023-02109_review_history.pdf]

Life Science Alliance

The highly improved genome of Ixodes scapularis with X and Y pseudochromosomes

Andrew Nuss, Jonathan Lomas, Jeremiah Reyes, Omar Garcia-Cruz, Wenlong Lei, Arvind Sharma, Michael Pham, Saransh Beniwal, Mia Swain, Molly McVicar, Isaac Hinne, Xingtan Zhang, Won Yim, and Monika Gulia-Nuss
DOI: https://doi.org/10.26508/lsa.202302109

Corresponding author(s): Monika Gulia-Nuss, University of Nevada Reno and Won Yim, Department of Biochemistry and Molecular Biology, the University of Nevada, Reno, USA

**Review Timeline:**		
	Submission Date:	2023-04-21
	Editorial Decision:	2023-05-21
	Revision Received:	2023-08-14
	Editorial Decision:	2023-09-07
	Revision Received:	2023-09-21
	Accepted:	2023-09-22

Scientific Editor: Novella Guidi

Transaction Report:

(Note: With the exception of the correction of typographical or spelling errors that could be a source of ambiguity, letters and reports are not edited. The original formatting of letters and referee reports may not be reflected in this compilation.)

May 21, 2023

Re: Life Science Alliance manuscript #LSA-2023-02109-T

Monika Gulia-Nuss
University of Nevada, Reno

Dear Dr. Gulia-Nuss,

Thank you for submitting your manuscript entitled "Highly improved reference genome of Ixodes scapularis with X and Y chromosomes" to Life Science Alliance. The manuscript was assessed by expert reviewers, whose comments are appended to this letter. We invite you to submit a revised manuscript addressing the Reviewer comments.

To upload the revised version of your manuscript, please log in to your account: https://lsa.msubmit.net/cgi-bin/main.plex

You will be guided to complete the submission of your revised manuscript and to fill in all necessary information. Please get in touch in case you do not know or remember your login name.

While you are revising your manuscript, please also attend to the below editorial points to help expedite the publication of your manuscript. Please direct any editorial questions to the journal office.

The typical timeframe for revisions is three months. Please note that papers are generally considered through only one revision cycle, so strong support from the referees on the revised version is needed for acceptance.

When submitting the revision, please include a letter addressing the reviewers' comments point by point.

We hope that the comments below will prove constructive as your work progresses.

Thank you for this interesting contribution to Life Science Alliance. We are looking forward to receiving your revised manuscript.

Sincerely,

Novella Guidi, PhD
Scientific Editor
Life Science Alliance

A. THESE ITEMS ARE REQUIRED FOR REVISIONS

-- A letter addressing the reviewers' comments point by point.

-- An editable version of the final text (.DOC or .DOCX) is needed for copyediting (no PDFs).

-- High-resolution figure, supplementary figure and video files uploaded as individual files: See our detailed guidelines for preparing your production-ready images, https://www.life-science-alliance.org/authors

-- Summary blurb (enter in submission system): A short text summarizing in a single sentence the study (max. 200 characters including spaces). This text is used in conjunction with the titles of papers, hence should be informative and complementary to the title and running title. It should describe the context and significance of the findings for a general readership; it should be written in the present tense and refer to the work in the third person. Author names should not be mentioned.

-- By submitting a revision, you attest that you are aware of our payment policies found here: https://www.life-science-alliance.org/copyright-license-fee

B. MANUSCRIPT ORGANIZATION AND FORMATTING:

Full guidelines are available on our Instructions for Authors page, https://www.life-science-alliance.org/authors

We encourage our authors to provide original source data, particularly uncropped/-processed electrophoretic blots and spreadsheets for the main figures of the manuscript. If you would like to add source data, we would welcome one PDF/Excel-file

per figure for this information. These files will be linked online as supplementary "Source Data" files.

IMPORTANT: It is Life Science Alliance policy that if requested, original data images must be made available. Failure to provide original images upon request will result in unavoidable delays in publication. Please ensure that you have access to all original microscopy and blot data images before submitting your revision.

Reviewer #1 (Comments to the Authors (Required)):

In the current manuscript, Nuss et al. report their efforts in developing a complete genome of Ixodes scapularis using multiple tick stages and sexes. The authors predicted the identity of 13 pseudochromosomes, as potential autosomes and, using a read-based phasing strategy, denoted 2 additional pseudo-chromosomes as possible sex chromosomes: X and Y. The authors also detailed parameters to present a markedly improved genome annotation. However, at its current stage, the manuscript presents insufficient description of some of the methodologies and evidence, and fails to include adequate comparisons and information from up-to-date literature. While the authors are commended for their use of male DNA and prediction of a chromosome-scale scaffold as being part of the Y chromosome, the manuscript features many deficiencies, as explained below. The following points are raised for consideration:

1. In the title and throughout the manuscript, the authors referred to "chromosome" (or at times as "autosomes" or "sex chromosomes" "X" or Y"). Without proper karyotyping and identification of chromosomes by FISH or relevant technologies using scaffold-specific terminal probes, the use of these nomenclatures seems like an overinterpretation, and should be labelled as chromosome-scale scaffolds or pseudochromosomes.

2. In the main figures, the authors should also compare the quality and improvement of their currently produced genome with the most updated reference genome, published early this year (De et al., A high-quality Ixodes scapularis genome advances tick science, Nature Genetics, 55(2):301-311. Epub Jan 19, 2023.). In the current study, these comparisons are mainly done with a fragmented genome with partial (57%) coverage (IscaW1) or other older genomes, including distant species.

3. The statement in Lines 256-257 (or page 269) "The size of our assembly is comparable to an unpublished I. scapularis genome assembly deposited into the NCBI GenBank under the accession name ASM1692078v2" needed to be updated. This assembly has been published (De et al., Nature Genetics, 55(2): 2023).

4. Did the authors check for the absence of common tick-borne pathogens in various stages or sexes of ticks, used for genome assembly? Has the non-Ixodes DNA been identified and removed from the assembly?

5. Please clarify the statement "IscGN being the first to be chromosome-scale" as a comparable chromosome-level I. scapularis genome is available (De et al., Nature Genetics, 55(2): 2023).

6. The current genome is estimated to be larger than the ASM1692078v2 assembly; the authors should explain possible reasons why additional increments (such as 474 Mb in the female assembly) exist in the genome. Unlike the published ASM1692078v2 assembly, which was primarily derived from HiFi sequencing of two haplotypes within one individual, the current assembly used multiple adults, and thus multiple haplotypes. Is it possible that these apparent increments are due to duplications, repeat sequences, or other possibilities? The author should compare the ASM1692078v2 with the current assembly.

7. The current genome assembly used PacBio HiFi sequencing and contigs derived from multiple male or female adults, while the scaffolding used CHiCAGO and Hi-C sequencing originated from egg masses. It is unclear why disparate tick stages were used as the DNA source for contig generation and scaffolding. Chromatin changes or remodeling are known to occur in the embryonic stages; it remains unclear whether this or similar DNA remodeling events would impact the correct scaffolding of HiFi contigs. Without sufficient evidence, whether the genome-wide chromatin interactions are preserved in eggs and adults, the Hi-C and HiFi sequencing should ideally involve the same tick stages for genome assembly, which should be addressed in the manuscript.

8. The authors need to provide more details on the improvement of the annotation and gene models in IscGN, particularly as compared to the recently developed NCBI reference genome (ASM1692078v2), which was annotated using full-length RNA isoform sequencing. As one example, in Table S1, the mean CDS length (bp) of the current annotation is 218.07, which is considerably lower than all previous gene models, which have means between 855-1,507 bp. The reason for these differences should be described more clearly so the readers can appreciate the improvement in the current genome.

9. The MAKER genome annotation pipeline, as well as many other platforms, were used to produce an annotation for the IscGN assembly. However, other than just referencing these platforms, additional relevant details are missing, such as any codes, major steps, key parameters, and detailed information; these should be included in the Method section or provided as supplementary information.

10. The core gene families were curated manually, which is acceptable. There is insufficient description of the methods and threshold parameters used to group such genes into a particular family, which should be included in the Method section.

11. While the identity of sex chromosome-scale scaffolds accounts for the most interesting data in the manuscript, it remains unclear how these were accomplished. The authors claim that these scaffolds were identified using a read-depth strategy that compared the alignment rate of sequencing reads generated from male and female ticks, and possibly identify the Y chromosome-scale scaffold, as detailed in Line 355, but there is no specific mention of how the X chromosome scaffold is identified. As conclusive evidence, the chromosome-level scaffolds that contain the SDR (sex determination region) or male-specific region of the Y (MSY), and if applicable the pseudoautosomal regions (PAR), should be labeled in the sex chromosome designation. Ideally, the genomic location of these elements should be included in the genome release.

12. The databases in which the assembly and annotation were deposited are missing?

13. The manuscript requires editing for typos. The following represents several examples:

a) Line 119 paragraph refers to Fig. 3. This seems to be incorrect, and should be Fig. 2.
b) Line 142: "chromosome Y has the highest TE content (Fig. 4; Supplementary Table 5)". This seems to be Fig. 3, not Fig. 4 as mentioned.
c) Line 144: "(LTR) retrotransposons, comprising 15.16% of the genome (Fig. 4;". This seems to be Fig. 3, not Fig. 4 as mentioned.
d) L23: Correct as "....470,000 estimated Lyme disease cases annually in the United States."
e) L28: should read as "I. scapularis"
f) L37: Without proper comparison, the statement "markedly improved genome annotation" should be modified.
g) L56: "However, even the newer tick genomes suffer from the same fragmentation issue.." It is unclear exactly what the authors are referring to. Newer tick genomes do not have the "same" fragmentation issues as older genomes. The statement should be modified accordingly.
h) Table S8: It is unclear what the subtitle meant ("Old data - need to update").
i) Cuticular proteins: It seems that the database was last updated on 20 Oct 2009. Does this still represent an up-to-date curation?
j) The figures lack sufficient resolution and are difficult to view and read.

Reviewer #2 (Comments to the Authors (Required)):

Nuss et al present a dramatically improved assembly of the Ixodes scapularis genome. Using state-of-the-art methods, they generated a pseudo-chromosomal level assembly which they then annotated used RNAseq data. While there is little in the way of analysis beyond gene family size, the study will no doubt be a foundation upon which future tick research is based on. The manuscript is clear and well written, the analyses are appropriate and well described, and the conclusions are well supported by their analyses. I only have minor comments on the manuscript.

The authors indicate that they annotated the genome using RNAseq data (line 128 - 131), but information on how these samples were generated (RNA extraction method, sequencing details) and what data resulted from them seem to be missing from the methods/supplemental results.

Figure 5 has a white box in the middle of it; presumably this is just an error when converting from word to PDF?

I did not see accession numbers for the I. scapularis assembly/annotation or the RNAseq data in the manuscript. If the journal allows it, it might be helpful to put this in a "data accessibility" section at the end of the manuscript or in the results section.

Table S8 says "Old data - need to update"

Table S11 - there are some random data in column 1 below the table.

Reviewer #3 (Comments to the Authors (Required)):

Authors assembly a high-quality genome of the important vector tick Ixodes scapularis using Hi-C, PacBio HiFi sequencing, and Illumina short-read sequencing technologies, which produced a chromosome level genome. They further assembled the X and Y chromosomes, and characterized genes belonging to chemosensory functions, proteases and protease inhibitors, and cuticular proteins, as well as the Hox cluster on Chromosome 1. The results shed lights on the understanding of the adaptation mechanisms of tick, and are of great significance for future control of ticks and tick-borne diseases. There are some concerns authors should pay attention:

1. Authors compared the assembled genome with previous published genomes, whereas two genomes were omitted, De et al., 2023 (A high-quality Ixodes scapularis genome advances tick science. Nat Genet 55, 301-311) and Yu et al. 2022 (The new Haemaphysalis longicornis genome provides insights into its requisite biological traits. Genomics, 114(2), 110317); More information should be provided if "the NCBI GenBank under the accession name ASM1692078v2 (Pal lab, unpublished)" refers to De et al., 2023.
2. Sample collection, more information should be provided for the origin of the "Fully engorged I. scapularis females", the generations, and the host, etc.
3. Methods for "Transcriptome analysis", more information should be provided, which stage or which tissues were used for sequencing;
4. Line 131, "eggs, larvae, nymph, and adult male and female" should use same plural forms;
5. Line 54, "Xiaofeng et al., 2018" seems not find in the reference list;
6. Reference list, full journal name or abbreviation should be uniform;
7. Fig. 1B, value in X-axis of ChrX was wrongly displayed

Reviewer #1 (Comments to the Authors (Required)):

In the current manuscript, Nuss et al. report their efforts in developing a complete genome of Ixodes scapularis using multiple tick stages and sexes. The authors predicted the identity of 13 pseudochromosomes, as potential autosomes and, using a read-based phasing strategy, denoted 2 additional pseudo-chromosomes as possible sex chromosomes: X and Y. The authors also detailed parameters to present a markedly improved genome annotation. However, at its current stage, the manuscript presents insufficient description of some of the methodologies and evidence, and fails to include adequate comparisons and information from up-to-date literature. While the authors are commended for their use of male DNA and prediction of a chromosome-scale scaffold as being part of the Y chromosome, the manuscript features many deficiencies, as explained below. The following points are raised for consideration:

1. In the title and throughout the manuscript, the authors referred to "chromosome" (or at times as "autosomes" or "sex chromosomes" "X" or Y"). Without proper karyotyping and identification of chromosomes by FISH or relevant technologies using scaffold-specific terminal probes, the use of these nomenclatures seems like an overinterpretation, and should be labelled as chromosome-scale scaffolds or pseudochromosomes.

We agree with the reviewer and have made suggested changes.

2. In the main figures, the authors should also compare the quality and improvement of their currently produced genome with the most updated reference genome, published early this year (De et al., A high-quality Ixodes scapularis genome advances tick science, Nature Genetics, 55(2):301-311. Epub Jan 19, 2023.). In the current study, these comparisons are mainly done with a fragmented genome with partial (57%) coverage (IscaW1) or other older genomes, including distant species.

We have revised the figures and text per the reviewers' valuable feedback. Additionally, we have now included a comprehensive comparison with the findings of De et al., 2023, as suggested by the reviewer.

3. The statement in Lines 256-257 (or page 269) "The size of our assembly is comparable to an unpublished I. scapularis genome assembly deposited into the NCBI GenBank under the accession name ASM1692078v2" needed to be updated. This assembly has been published (De et al., Nature Genetics, 55(2): 2023).

We have ensured that the citation is included in the appropriate section of the text, allowing readers and reviewers to access the relevant source for further context and validation of our research.

4. Did the authors check for the absence of common tick-borne pathogens in various stages or sexes of ticks, used for genome assembly? Has the non-Ixodes DNA been identified and removed from the assembly?

To clarify, the DNA extraction process involved using whole ticks after surface cleaning. It is important to note that these ticks are pathogen-free, as they are part of our maintained colony. While we did not conduct specific pathogen testing on the ticks for this particular study, it is worth mentioning that pathogen screening is routinely incorporated into our rearing protocol. We

randomly select tick samples from the colony for testing to ensure that known pathogens are not present.

Additionally, before proceeding with the assembly, we employed Centrifuge (Kim et al., 2016) to perform DNA decontamination, aiming to identify and remove any non-tick DNA contaminants from the samples. Despite conducting the decontamination process, no contaminations were detected, resulting in the decision to omit a detailed explanation of this step in the manuscript.

5. Please clarify the statement "IscGN being the first to be chromosome-scale" as a comparable chromosome-level I. scapularis genome is available (De et al., Nature Genetics, 55(2): 2023).

This statement has been modified.

6. The current genome is estimated to be larger than the ASM1692078v2 assembly; the authors should explain possible reasons why additional increments (such as 474 Mb in the female assembly) exist in the genome. Unlike the published ASM1692078v2 assembly, which was primarily derived from HiFi sequencing of two haplotypes within one individual, the current assembly used multiple adults, and thus multiple haplotypes. Is it possible that these apparent increments are due to duplications, repeat sequences, or other possibilities? The author should compare the ASM1692078v2 with the current assembly.

A thorough comparison has been conducted between our findings and De et al. 2023 findings. The observed difference of approximately 200 Mb in chromosome-scale comparison could be attributed to variations in repeat sequences or differences among the strains analyzed in both studies. These factors may account for the observed distinctions in the genome sizes between the two datasets.

Furthermore, our investigation revealed that the 200Mb differences primarily stem from additional repeat sequences, indicating a surplus of 155Mbp of repeat contents compared to De et al. These findings have been elucidated and discussed in detail in the dedicated discussion section of our manuscript (Lines 327-336).

7. The current genome assembly used PacBio HiFi sequencing and contigs derived from multiple male or female adults, while the scaffolding used CHiCAGO and Hi-C sequencing originated from egg masses. It is unclear why disparate tick stages were used as the DNA source for contig generation and scaffolding. Chromatin changes or remodeling are known to occur in the embryonic stages; it remains unclear whether this or similar DNA remodeling events would impact the correct scaffolding of HiFi contigs. Without sufficient evidence, whether the genome-wide chromatin interactions are preserved in eggs and adults, the Hi-C and HiFi sequencing should ideally involve the same tick stages for genome assembly, which should be addressed in the manuscript.

Our approach involved utilizing egg masses for HiC in an effort to enhance the existing IscaW genome assembly, which originally stemmed from eggs. Despite employing HiC/HiRise assembly, the resulting output still consisted of 83,347 scaffolds, a significant improvement compared to the 369,495 scaffolds present in the IscaW assembly (Nuss et al., BioRxiv), but the assembly was still not at the C-scaffolds level.

To further enhance contiguity and potentially identify phased sex chromosomes, we pursued PacBio sequencing using male (pool of 5) and female (1) high-molecular-weight (HMW) DNA.

Subsequently, we reran the HiRise assembly, incorporating these new DNA sequences, which yielded 14 C-scaffolds indicative of chromosome-scale genome scaffolds.

While we acknowledge that the higher-order chromatin organization of eggs and adults might differ; yet, in Hi-C scaffolding, the choice of materials is less important because it targets the reconstruction of the whole genome as the uniform goal, even when using different cell populations in an organism. Existing research has suggested that incorporating diverse types of tissues in Hi-C studies can yield optimal results, capturing a broader spectrum of chromatin contacts.

8. The authors need to provide more details on the improvement of the annotation and gene models in IscGN, particularly as compared to the recently developed NCBI reference genome (ASM1692078v2), which was annotated using full-length RNA isoform sequencing. As one example, in Table S1, the mean CDS length (bp) of the current annotation is 218.07, which is considerably lower than all previous gene models, which have means between 855-1,507 bp. The reason for these differences should be described more clearly so the readers can appreciate the improvement in the current genome.

Thank you for bringing this matter to our attention; we sincerely appreciate your vigilance in ensuring the accuracy and integrity of our research. Reviewing our previous results, we identified an error in the reported mean CDS length. To rectify this, we have updated the data in Supplementary Table 1 with the correct values to 1,386 bp.

9. The MAKER genome annotation pipeline, as well as many other platforms, were used to produce an annotation for the IscGN assembly. However, other than just referencing these platforms, additional relevant details are missing, such as any codes, major steps, key parameters, and detailed information; these should be included in the Method section or provided as supplementary information.

We understand the importance of providing detailed and comprehensive methods to ensure clarity and avoid potential confusion among researchers. To address this concern, we have thoroughly revised the methods section, incorporating additional details and step-by-step explanations of the procedures employed in our study.

10. The core gene families were curated manually, which is acceptable. There is insufficient description of the methods and threshold parameters used to group such genes into a particular family, which should be included in the Method section.

Thank you for bringing up the lack of details. We have thoroughly revised the methods section to address this concern, incorporating additional information and threshold parameters employed in our study.

11. While the identity of sex chromosome-scale scaffolds accounts for the most interesting data in the manuscript, it remains unclear how these were accomplished. The authors claim that these scaffolds were identified using a read-depth strategy that compared the alignment rate of sequencing reads generated from male and female ticks and possibly identify the Y chromosome-scale scaffold, as detailed in Line 355, but there is no specific mention of how the X chromosome scaffold is identified. As conclusive evidence, the chromosome-level scaffolds that contain the SDR (sex determination region) or male-specific region of the Y (MSY), and if

applicable, the pseudoautosomal regions (PAR), should be labeled in the sex chromosome designation. Ideally, the genomic location of these elements should be included in the genome release.

We have taken the necessary steps to provide additional details regarding identifying the X-chromosome scaffold (Line 427-441). Through our analysis, we identified pseudo sex chromosomes or their specific segments by analyzing the remarkable disparity in read coverage between male and female samples.

The analysis of chromosome depth and sequencing coverage can provide valuable insights into the accuracy of the chromosome assignment (Line 275-298).

As advancements in sequencing technology and data accumulation continue, the possibility of identifying the MSY based on K-mers will likely increase. To further enhance our understanding of the genome's structure, we plan to identify male-specific sequences. This will involve conducting Fluorescence In Situ Hybridization (FISH) experiments to localize specific DNA sequences within the genome.

12. The databases in which the assembly and annotation were deposited are missing?

The genome sequence has been successfully uploaded to the NCBI database under the accession number JAMZAT000000000, and it is associated with BioSample SAMN26733336. However, please note that the data is currently in an embargo state. Once our manuscript is accepted for publication, we are committed to promptly releasing the genome sequences along with comprehensive annotation. Our priority is to ensure open access to the genomic data, enabling other researchers to validate and build upon our findings.

13. The manuscript requires editing for typos. The following represents several examples:

a) Line 119 paragraph refers to Fig. 3. This seems to be incorrect, and should be Fig. 2.
b) Line 142: "chromosome Y has the highest TE content (Fig. 4; Supplementary Table 5)". This seems to be Fig. 3, not Fig. 4 as mentioned.
c) Line 144: "(LTR) retrotransposons, comprising 15.16% of the genome (Fig. 4;". This seems to be Fig. 3, not Fig. 4 as mentioned.
d) L23: Correct as "....470,000 estimated Lyme disease cases annually in the United States."
e) L28: should read as "I. scapularis"
f) L37: Without proper comparison, the statement "markedly improved genome annotation" should be modified.
g) L56: "However, even the newer tick genomes suffer from the same fragmentation issue.." It is unclear exactly what the authors are referring to. Newer tick genomes do not have the "same" fragmentation issues as older genomes. The statement should be modified accordingly.
All the errors mentioned in a-g have been corrected.

h) Table S8: It is unclear what the subtitle meant ("Old data - need to update").
Our apologies for this oversight. The table has been modified, and old data has been removed.

i) Cuticular proteins: It seems that the database was last updated on 20 Oct 2009. Does this still represent an up-to-date curation?
Thank you for the suggestion. We have updated these data with a newer database version.
j) The figures lack sufficient resolution and are difficult to view and read.

We will submit high-resolution images.

Reviewer #2 (Comments to the Authors (Required)):

Nuss et al present a dramatically improved assembly of the Ixodes scapularis genome. Using state-of-the-art methods, they generated a pseudo-chromosomal level assembly which they then annotated used RNAseq data. While there is little in the way of analysis beyond gene family size, the study will no doubt be a foundation upon which future tick research is based on. The manuscript is clear and well written, the analyses are appropriate and well described, and the conclusions are well supported by their analyses. I only have minor comments on the manuscript.

The authors indicate that they annotated the genome using RNAseq data (line 128 - 131), but information on how these samples were generated (RNA extraction method, sequencing details) and what data resulted from them seem to be missing from the methods/supplemental results.

RNAseq methods have been added.

Figure 5 has a white box in the middle of it; presumably this is just an error when converting from word to PDF?

I did not see accession numbers for the I. scapularis assembly/annotation or the RNAseq data in the manuscript. If the journal allows it, it might be helpful to put this in a "data accessibility" section at the end of the manuscript or in the results section.
The bioproject numbers have been added.

Table S8 says "Old data - need to update"
Our apologies for this oversight. The table has been modified, and old data has been removed.

Table S11 - there are some random data in column 1 below the table.
Our apologies for this oversight. The table has been modified.

Reviewer #3 (Comments to the Authors (Required)):

Authors assembly a high-quality genome of the important vector tick Ixodes scapularis using Hi-C, PacBio HiFi sequencing, and Illumina short-read sequencing technologies, which produced a chromosome level genome. They further assembled the X and Y chromosomes, and characterized genes belonging to chemosensory functions, proteases and protease inhibitors, and cuticular proteins, as well as the Hox cluster on Chromosome 1. The results shed lights on the understanding of the adaptation mechanisms of tick, and are of great significance for future control of ticks and tick-borne diseases. There are some concerns authors should pay attention:

1. Authors compared the assembled genome with previous published genomes, whereas two genomes were omitted, De et al., 2023 (A high-quality Ixodes scapularis genome advances tick science. Nat Genet 55, 301-311) and Yu et al. 2022 (The new Haemaphysalis longicornis genome provides insights into its requisite biological traits. Genomics, 114(2), 110317); More information should be provided if "the NCBI GenBank under the accession name ASM1692078v2 (Pal lab, unpublished)" refers to De et al., 2023.

All of these errors have been fixed.

2. Sample collection, more information should be provided for the origin of the "Fully engorged I. scapularis females", the generations, and the host, etc.
3. Methods for "Transcriptome analysis", more information should be provided, which stage or which tissues were used for sequencing;
4. Line 131, "eggs, larvae, nymph, and adult male and female" should use same plural forms;
5. Line 54, "Xiaofeng et al., 2018" seems not find in the reference list;
6. Reference list, full journal name or abbreviation should be uniform;
7. Fig. 1B, value in X-axis of ChrX was wrongly displayed

Thank you for the suggestions. All of these errors have been fixed.

September 7, 2023

RE: Life Science Alliance Manuscript #LSA-2023-02109-TR

Dr. Monika Gulia-Nuss
University of Nevada Reno
Department of Biochemistry and Molecular Biology
1664 N Virginia st
Reno 89557

Dear Dr. Gulia-Nuss,

Thank you for submitting your revised manuscript entitled "The highly improved genome of Ixodes scapularis with X and Y pseudochromosomes". We would be happy to publish your paper in Life Science Alliance pending final revisions necessary to meet our formatting guidelines.

Along with points mentioned below, please tend to the following:
-please address Reviewer 1's remaining comments
-please upload your main manuscript text as an editable doc file
-please upload all figure files as individual ones; all figure legends should only appear in the main manuscript file
-please upload one figure per file
-titles in the system and the manuscript file must match
-please make sure the author order in your manuscript and our system match
-please add ORCID ID for the secondary corresponding author--they should have received instructions on how to do so
-please add your main figure and table legends to the main manuscript text after the references section
-please use the [10 author names et al.] format in your references (i.e., limit the author names to the first 10)
-there is a callout for Figure 5F, and Figure 5 has only A and B panels; please correct
-please add callouts for Figure 1A, C-H to your main manuscript text

If you are planning a press release on your work, please inform us immediately to allow informing our production team and scheduling a release date.

LSA now encourages authors to provide a 30-60 second video where the study is briefly explained. We will use these videos on social media to promote the published paper and the presenting author (for examples, see https://twitter.com/LSAjournal/timelines/1437405065917124608). Corresponding or first-authors are welcome to submit the video. Please submit only one video per manuscript. The video can be emailed to contact@life-science-alliance.org

To upload the final version of your manuscript, please log in to your account: https://lsa.msubmit.net/cgi-bin/main.plex
You will be guided to complete the submission of your revised manuscript and to fill in all necessary information. Please get in touch in case you do not know or remember your login name.

To avoid unnecessary delays in the acceptance and publication of your paper, please read the following information carefully.

A. FINAL FILES:

These items are required for acceptance.

-- An editable version of the final text (.DOC or .DOCX) is needed for copyediting (no PDFs).

-- High-resolution figure, supplementary figure and video files uploaded as individual files: See our detailed guidelines for preparing your production-ready images, https://www.life-science-alliance.org/authors

-- Summary blurb (enter in submission system): A short text summarizing in a single sentence the study (max. 200 characters including spaces). This text is used in conjunction with the titles of papers, hence should be informative and complementary to the title. It should describe the context and significance of the findings for a general readership; it should be written in the present tense and refer to the work in the third person. Author names should not be mentioned.

B. MANUSCRIPT ORGANIZATION AND FORMATTING:

Full guidelines are available on our Instructions for Authors page, https://www.life-science-alliance.org/authors

We encourage our authors to provide original source data, particularly uncropped/-processed electrophoretic blots and spreadsheets for the main figures of the manuscript. If you would like to add source data, we would welcome one PDF/Excel-file per figure for this information. These files will be linked online as supplementary "Source Data" files.

Submission of a paper that does not conform to Life Science Alliance guidelines will delay the acceptance of your manuscript.

It is Life Science Alliance policy that if requested, original data images must be made available to the editors. Failure to provide original images upon request will result in unavoidable delays in publication. Please ensure that you have access to all original data images prior to final submission.

The license to publish form must be signed before your manuscript can be sent to production. A link to the electronic license to publish form will be sent to the corresponding author only. Please take a moment to check your funder requirements.

Reviews, decision letters, and point-by-point responses associated with peer-review at Life Science Alliance will be published online, alongside the manuscript. If you do want to opt out of having the reviewer reports and your point-by-point responses displayed, please let us know immediately.

Thank you for your attention to these final processing requirements. Please revise and format the manuscript and upload materials within 7 days.

Thank you for this interesting contribution, we look forward to publishing your paper in Life Science Alliance.

Sincerely,

Novella Guidi, PhD
Scientific Editor
Life Science Alliance

Reviewer #1 (Comments to the Authors (Required)):

This is an improved version of the paper. The authors positively responded to the criticisms raised in the previous version of the manuscript, adding new information and data analysis that further strengthen their conclusions. Nevertheless, I have a few remaining concerns that need to be addressed:

Line 427-441: The additional details, as indicated, regarding the identification of the X-chromosome scaffold, are still insufficient. There is a generic description of contig characterization, but specifically how the X-chromosome scaffold is identified is still not clear. Given the importance of the topic, absence of FISH data, and as the concerned scaffold is often marked as "chromosome" (such as line 67, 75 etc.), this issue should be clarified in the revised manuscript.

Fig 2: To avoid confusion, the authors should separate (or clearly indicate) panel A, which shows 15 pseudomolecules, not the complete (585) scaffold set, which were otherwise represented in panels B-D.

Line 273-275: The statement "whereas the Pal lab assembly ...from BEI resources" seems to be incorrect. As the De et al (2023) paper indicates, the hatched egg masses were purchased from OSU Tick Rearing Facility and then reared in the Pal laboratory for several months, though the larval-nymphal as well as nymphal-adult molts, before use in the study.

Reviewer #1:

This is an improved version of the paper. The authors positively responded to the criticisms raised in the previous version of the manuscript, adding new information and data analysis that further strengthen their conclusions.

Thank you for the positive review of the revised manuscript.

Nevertheless, I have a few remaining concerns that need to be addressed:

Line 427-441: The additional details, as indicated, regarding the identification of the X-chromosome scaffold, are still insufficient. There is a generic description of contig characterization, but specifically how the X-chromosome scaffold is identified is still not clear. Given the importance of the topic, absence of FISH data, and as the concerned scaffold is often marked as "chromosome" (such as line 67, 75 etc.), this issue should be clarified in the revised manuscript.

The current assembly, constructed using sophisticated computational algorithms and cutting-edge sequencing technology, offers a high-confidence model of the tick genome. However, we concur with the reviewers that supplementing our bioinformatically derived genome assembly and pseudochromosome designations with FISH data is critical for unambiguous chromosomal assignments. We have added information to make the X-scaffold identification clear. Specifically, Lines 348-352 expound upon the concept of sex pseudochromosome phasing, while Lines 436-444 elaborate in detail on the methodology used to identify the X-chromosome scaffold. To address the reviewer's concern about the term 'chromosome' for sex chromosome, the manuscript was revised to explicitly state that the designations should be considered 'pseudochromosomes' until substantiated by FISH or other experimental validations. We have edited the whole manuscript to avoid calling it “chromosomes”.

Fig 2: To avoid confusion, the authors should separate (or clearly indicate) panel A, which shows 15 pseudomolecules, not the complete (585) scaffold set, which were otherwise represented in panels B-D.

Thank you for the suggestion. To keep the data consistent, we have used all 585 scaffolds.

Line 273-275: The statement "whereas the Pal lab assembly ...from BEI resources" seems to be incorrect. As the De et al (2023) paper indicates, the hatched egg masses were purchased from OSU Tick Rearing Facility and then reared in the Pal laboratory for several months, though the larval-nymphal as well as nymphal-adult molts, before use in the study.

We apologize for overlooking this information. We have now corrected this in the manuscript (Line 276-277)

September 22, 2023

RE: Life Science Alliance Manuscript #LSA-2023-02109-TRR

Dr. Monika Gulia-Nuss
University of Nevada Reno
Department of Biochemistry and Molecular Biology
1664 N Virginia st
Reno 89557

Dear Dr. Gulia-Nuss,

Thank you for submitting your Resource entitled "The highly improved genome of Ixodes scapularis with X and Y pseudochromosomes". It is a pleasure to let you know that your manuscript is now accepted for publication in Life Science Alliance. Congratulations on this interesting work.

The final published version of your manuscript will be deposited by us to PubMed Central upon online publication.

Your manuscript will now progress through copyediting and proofing. It is journal policy that authors provide original data upon request.

Reviews, decision letters, and point-by-point responses associated with peer-review at Life Science Alliance will be published online, alongside the manuscript. If you do want to opt out of having the reviewer reports and your point-by-point responses displayed, please let us know immediately.

IMPORTANT: If you will be unreachable at any time, please provide us with the email address of an alternate author. Failure to respond to routine queries may lead to unavoidable delays in publication.

Scheduling details will be available from our production department. You will receive proofs shortly before the publication date. Only essential corrections can be made at the proof stage so if there are any minor final changes you wish to make to the manuscript, please let the journal office know now.

DISTRIBUTION OF MATERIALS:
Authors are required to distribute freely any materials used in experiments published in Life Science Alliance. Authors are encouraged to deposit materials used in their studies to the appropriate repositories for distribution to researchers.

You can contact the journal office with any questions, contact@life-science-alliance.org

Again, congratulations on a very nice paper. I hope you found the review process to be constructive and are pleased with how the manuscript was handled editorially. We look forward to future exciting submissions from your lab.

Sincerely,

Novella Guidi, PhD
Scientific Editor
Life Science Alliance
